# Simultaneous quantitative analysis and *in vitro* anti-arthritic effects of five polyphenols from *Terminalia chebula*

**DOI:** 10.3389/fphys.2023.1138947

**Published:** 2023-03-08

**Authors:** Fang Liu, Shipeng Zhan, Pu Zhang, Changsheng Jia, Qingzong Zhu, Qing Dai, Mingjie Yu, Lin Cheng, Lirong Xiong, Fengjun Sun, Peiyuan Xia, Xiao Zhang, Jing Hu

**Affiliations:** ^1^ Department of Pharmacy, Southwest Hospital, Third Military Medical University (Army Medical University), Chongqing, China; ^2^ NMPA Key Laboratory for Quality Monitoring of Narcotic Drugs and Psychotropic Substances, Chongqing Institute for Food and Drug Control, Chongqing, China; ^3^ Institute of Pathology and Southwest Cancer Center, Southwest Hospital, Third Military Medical University (Army Medical University), Chongqing, China

**Keywords:** *Terminalia chebula*, simultaneous quantitative analysis, DPPH assay, anti-arthritic effect, principal components analysis

## Abstract

**Background:** The fruit of *Terminalia chebula* has been widely used for a thousand years for treating diarrhea, ulcers, and arthritic diseases in Asian countries. However, the active components of this Traditional Chinese medicine and their mechanisms remain unclear, necessitating further investigation.

**Objectives:** To perform simultaneous quantitative analysis of five polyphenols in *T. chebula* and evaluate their anti-arthritic effects including antioxidant and anti-inflammatory activity *in vitro*.

**Materials and methods:** Water, 50% water-ethanol, and pure ethanol were used as extract solvents. Quantitative analysis of gallic acid, corilagin, chebulanin, chebulagic acid, and ellagic acid in the three extracts was performed using high-performance liquid chromatography (HPLC). Antioxidant activity was assessed by the 2,2-diphenylpicrylhydrazyl (DPPH) radical-scavenging assay, and anti-inflammatory activity was evaluated by detecting interleukin (IL)-6 and IL-8 expression in IL-1β-stimulated MH7A cells.

**Results:** The 50% water-ethanol solvent was the optimal solvent yielding the highest total polyphenol content, and the concentrations of chebulanin and chebulagic acid were much higher than those of gallic acid, corilagin, and ellagic acid in the extracts. The DPPH radical-scavenging assay showed that gallic acid and ellagic acid were the strongest antioxidative components, while the other three components showed comparable antioxidative activity. As for the anti-inflammatory effect, chebulanin and chebulagic acid significantly inhibited IL-6 and IL-8 expression at all three concentrations; corilagin and ellagic acid significantly inhibited IL-6 and IL-8 expression at high concentration; and gallic acid could not inhibit IL-8 expression and showed weak inhibition of IL-6 expression in IL-1β-stimulated MH7A cells. Principal component analysis indicated that chebulanin and chebulagic acid were the main components responsible for the anti-arthritic effects of *T. chebula*.

**Conclusion:** Our findings highlight the potential anti-arthritic role of chebulanin and chebulagic acid from *T. chebula*.

## 1 Introduction

Rheumatoid arthritis (RA) is a complex chronic and systematic autoimmune disease that is characterized by synovial cell hyperplasia, consecutive inflammation, and progressive joint destruction, eventually leading to considerable disability (Weyand and Goronzy, 2021). Synovial cells, T cells, and macrophages are the three main immune cell types in RA disease progression (Jang et al., 2022). Among them, activated synovial cells are mainly involved in the pathogenesis and progression of RA, and pro-inflammatory mediators such as tumor necrosis factor (TNF)-α and interleukin (IL)-1β are typical activators of synovial cells (Ridgley et al., 2018; Chen et al., 2019). In addition to stimulating their own production, these mediators also induce the production of other inflammatory cytokines and mediators, including IL-6 and IL-8, and increased levels of these inflammatory mediators induce persistent inflammation and cartilage degradation (McInnes et al., 2016). Therefore, inhibition of the production of inflammatory cytokines by activated synovial cells is considered a potential therapeutic target for arthritis treatment. Disease-modifying anti-rheumatic drugs (DMARDs) are mainly used in clinical practice and include conventionally synthesized DMARDs, biological DMARDs, and targeted synthetic DMARDs (Singh et al., 2016; Smolen et al., 2020). The guidelines recommend conventionally synthesized DMARDs, especially methotrexate, as the first choice for RA treatment, but long-term use of these drugs may cause adverse reactions such as gastrointestinal lesions, liver toxicity, and cardiovascular complications, leading to intolerance (Singh et al., 2016). Biological MDARDs and targeted synthetic DMARDs are newly developed drugs with promising results, but approximately 20% of the patients still do not show good responses to those drugs. Moreover, those drugs are expensive, and their long-term use may cause adverse reactions such as respiratory tract infection and blood system toxicity. Therefore, development of new drugs for the treatment of RA has remained a key objective.

The fruit of *Terminalia chebula* Retz has been widely used as a folk medicine in India, China, and other Asian countries (Nigam et al., 2020). In traditional Chinese medicine, especially in Tibetan and Mongolian medicine, *T. chebula* is known as the “king of medicines” because of its extraordinary range of anticancer, antioxidative, antidiabetic, anti-inflammatory, antimicrobial, and immunomodulatory activities (Zhou et al., 2017; He et al., 2021). The antiarthritic effects and ability to relieve joint discomfort of water and ethanol extracts of *T. chebula* have been examined in animal models and clinical studies (Kalaiselvan and Rasool, 2015; Karlapudi et al., 2018; Ekambaram et al., 2020). Previous phytochemical studies indicated that polyphenols, flavonoids and triterpenoids are the main effective components of these extracts (Gowda et al., 2012; Hedina et al., 2016). In our previous study, we conducted a network-based pharmacology analysis and screened out 10 potential active components (six polyphenols and four alkaloids) of *T. chebula* as the candidate active components for the treatment of RA (Fang et al., 2019). However, quantitative analysis of those polyphenols in *T. chebula* has not been performed and needs further investigation. Some studies evaluated the anti-inflammatory effect of chebulagic acid, one of polyphenols isolated from *T. chebula*, in LPS-stimulated RAW264.7 cell lines (Liu et al., 2015). However, the anti-inflammatory effects of polyphenols on inflammatory cytokine production by IL-1β-stimulated synovial cells have not been evaluated yet. MH7A is a novel immortalized rheumatoid fibroblast-like synoviocyte line that was established by stably transfecting FLS cells with the SV40 T antigen gene (Miyazawa et al., 1998). It is usually used for investigating the regulation of rheumatoid FLS to develop future RA therapy (Zhang et al., 2019; Kong et al., 2020). Studies on natural compounds’ anti-arthritic effects have mainly focused on their influence on antioxidant and anti-inflammatory effects (Yu et al., 2021). Therefore, in the present study, we quantitatively determined the polyphenols in different extracts and investigated the antioxidant effects of polyphenols by DPPH radical-scavenging assay, and further investigated the anti-inflammatory effect on the production of inflammatory cytokines such as IL-6 and IL-8 by IL-1β-stimulated synovial cells. The results from principal component analysis indicated that among those polyphenols, chebulanin and chebulagic acid are the main anti-arthritic components.

## 2 Materials and methods

### 2.1 Reagents

Chebulanin (cat. no. BCP28247, purity ≥ 95%) was purchased from Hanxiang Biotechnology Company (Shanghai, China); gallic acid (cat. no. BCP17127, purity ≥ 98%), corilagin (cat. no. F0304AS, purity ≥ 95%), chebulagic acid (cat. no. MB5881, purity ≥ 95%), and ellagic acid (cat. no. M0104AS, purity ≥ 98%) were purchased from Meilun biotechnology company (Dalian, China). Folin–Ciocalteu reagent (cat. no. 101625012) and 2,2-diphenylpicrylhydrazyl (DPPH; cat. no. 101285784) were supplied by Sigma-Aldrich Co. (St Louis, MO, United States). HPLC-grade methanol and acetonitrile were obtained from Dikma Technologies (Shanghai, China); fetal bovine serum (FBS) and Dulbecco’s modified Eagle’s medium (DMEM) were purchased from Gibco (New York, United States). Cell counting kit-8 (CCK-8) (cat. no. BG0025) was obtained from Bioground Company (Chongqing, China). Human IL-6 (cat. no. EK0410) and IL-8 (cat. no. EK0413) enzyme-linked immunosorbent assay (ELISA) kits were purchased from Boster Biological Technology Co., Ltd., (Wuhan, China). Recombinant human IL-1β (cat. no. 201-LB-005/CF) was purchased from R&D Systems (St Paul, MN).

### 2.2 Plant materials

*T. chebula* (simmer *T. chebula*, cat. no. 180605) originating from Guangxi Province was purchased from Chongqing Shangyao Huiyuan Pharmaceutical Co., (China) and authenticated by Prof. Fan Weiqiang, an expert in Tibetan medicine from Aba in Sichuan Institute for Food and Drug Control, People’s Republic of China.

### 2.3 Preparation of extracts

*T. chebula* was crushed into powder, and 50 g of the powder was extracted three times with 300 mL of water, 300 mL of 50% water-ethanol, or 300 mL of ethanol for 20 min by ultrasonication. The extracts from the three rounds of extraction were combined and filtered through a filter paper. The water extracts were lyophilized to obtain *chebula* water extract (CWE); the 50% water-ethanol and ethanol extracts were evaporated under pressure and then lyophilized, yielding *chebula* water-ethanol extract (CWEE) and *chebula* ethanol extract (CEE), respectively.

### 2.4 Total phenolic content determination

The total phenolic content of the extracts was determined by the Folin–Ciocalteu assay with some modification (Ainsworth and Gillespie, 2007). Briefly, 50 μL of Folin–Ciocalteu reagent (2N) was added to 100 μL of the extract solution and vortexed for 30 s, followed by the addition of 100 μL of 10% (w/v) sodium carbonate and vortexing for 30 s, incubation in the dark for 30 min, and measurement of absorbance at 760 nm. The total phenolic content was calculated from the calibration curve, and the results were expressed as mg of gallic acid equivalent per g of dry weight.

### 2.5 Simultaneous qualitative and quantitative analysis of polyphenols

We first used an AB SCIEX X500R Q-TOF high-resolution mass spectrometer to qualitatively analyze the components of the 50% water-ethanol extract. Next, Agilent ZORBAX Eclipse Plus C18 (2.1 mm × 50 mm, 1.8 μm) was used as the separation column; the mobile phase consisted of methanol (phase A) and 0.1% formic acid aqueous solution (phase B). The gradient elution was as follows: 0–3 min, 20%–90% B; 3–7 min, 90% B; 7–10 min, 10% B; flow rate was 0.3 mL/min, and the injection volume was 5 μL. Mass spectrometry analyses were performed with an electrospray ion source in a negative ion mode and the following parameters: ion source gas pressure, 45 psi; curtain gas pressure, 35 psi; temperature, 400°C; scan time, 10 min; electrospray voltage, −4500 V; declustering potential (DP), −80 V; and collision energy (CE), −10 V.

Then, we established and validated a rapid and sensitive HPLC method for simultaneous quantitative analysis of the polyphenols in different extracts. An HPLC system including a Waters 2695 Alliance peristaltic pump and a Waters 996D UV detector was employed for simultaneous quantitative analysis in this study. The separation column was Ultimate LP C18 (250 mm × 4.6 mm; particle size: 5 μm; Welch, Shanghai, China). The mobile phase consisted of methanol (phase A) and water containing 0.6% phosphoric acid (phase B) with the following gradient system: 5% (A) in 0–2 min, 5%–20% (A) in 2–10 min, 20%–27% (A) in 10–25 min, 27%–45% (A) in 25–40 min, 45%–100% (A) in 40–45 min, and 100%–5% (A) in 45–50 min. The flow rate was 1.2 mL/min using detection wavelengths of 278 nm and an injection volume of 10 μL.

### 2.6 Validation of the quantitative analyzed method

The validation of the method for simultaneous quantitative analyze of gallic acid, corilagin, chebulanin, chebulagic acid, and ellagic acid was conducted according to the International Conference on Harmonization (ICH) guidelines, ICH Q2 (R1) (Kumar et al., 2020). Specificity, linearity, range, accuracy, precision and stability were selected as validated parameters. The method’s specificity was determined by comparing the chromatograms obtained from the analysis of a blank, mixed standard solution, and sample solution. Linearity and range were performed by calibration curve of six concentrations of working standard solution. The *r* ^2^ of all curves were calculated. Accuracy was performed by determining low, medium and high concentrations of standard solution. The accuracy was shown as % recovery. Precision was conducted by analysis of standard solution at low, medium and high concentration. Intra-precision was calculated from parallel measurement of five copies within 1 day and inter-precision was calculated from parallel measurement of five copies over three consecutive days. The precision was shown as %RSD. Stability was conducted by analysis of test samples at 0 and 12 h respectively and shown as ratio between 0 and 12 h.

### 2.7 Antioxidant analysis

#### 2.7.1 DPPH radical-scavenging assay

Scavenging of DPPH free radicals is a common antioxidant assay. We assessed the DPPH free radical-scavenging activity of the extracts and individual components according to a published method with some modifications (Gulcin, 2020). Briefly, 100 μL of DPPH solution (100 μg/mL) was added to 100 μL of each extract and individual components of different concentrations (2, 5, 10, 25, 50, 125, and 250 μg/mL) and mixed gently for 30 s. The mixture was then allowed to incubate at room temperature for 30 min and the absorbance was determined at 518 nm and noted as A_i_. Absorbance of DPPH was also determined and noted as A_t_. The scavenging effect was calculated using the following formula:

$$DPPH-scavenging\;effect\;\left(\%\right)=\frac{At-Ai}{At}\times 100\%$$

### 2.8 Anti-inflammatory analysis

#### 2.8.1 Cell culture

Human rheumatoid arthritis synovial MH7A cells were obtained from RIKEN BRC cell bank and cultured in DMEM medium supplemented with 10% FBS at 37°C in 5% CO_2_ incubator. The cells were sub-cultured when cell fusion was above 85%.

#### 2.8.2 Assessment of cell viability by the CCK-8 assay

Cell viability was assessed by the CCK-8 assay. Briefly, MH7A cells were cultured in 96-well plates at 200 μL per well and a density of 5 × 10^3^ cells/well, different concentrations of the five polyphenols were added, and the cells were incubated for 24 h. Next, 20 μL of CCK-8 was added to each well and incubated at 37°C atmosphere for 1 h. The absorbance was measured at 450 nm.

#### 2.8.3 Determination of IL-6 and IL-8 expression by ELISA kits

MH7A cells were cultured in 96-well plates at a density of 5 × 10^3^ cells/well and pretreated with various concentrations of 50% water-ethanol extracts and individual polyphenols for 2 h. Then after, incubation with or without of IL-1β (5 ng/mL) for 6 h, the culture supernatants were collected and the concentrations of IL-6 and IL-8 were measured using ELISA kits according to the manufacturer’s instructions. The absorbance was measured at 450 nm and the concentration was calculated from a standard curve.

### 2.9 Principal component analysis

We used principal component analysis to rank the polyphenols and thereby identify the main active components. The contents of each polyphenol in the extracts, the IC_50_ values for DPPH-scavenging activity, and the IL-6 and IL-8 inhibition rates were selected as assessment indicators.

### 2.10 Statistical analysis

All statistical analysis were performed in IBM SPSS software version 25.0 (IBM Corp, Armonk, NY, United States). The IC_50_ values for the DPPH-scavenging effect were calculated by GraphPad software. All experimental values were expressed as mean ± standard deviation. Significant differences among multiple groups were determined by one-way analysis of variance, and *post-hoc* tests were conducted by Dunnett’s comparisons; *p*-values less than 0.05 were considered significant.

## 3 Results

### 3.1 Simultaneous qualitative and quantitative analysis of polyphenols in different extraction solvents

First, we utilized UPLC-Q-TOF technology for qualitative analysis of the components in 50% water-ethanol extract, and the results revealed six polyphenols (gallic acid, chebulic acid, corilagin, chebulanin, chebulagic acid, and ellagic acid) while four alkaloids were not identified, indicating that polyphenols may be the main components in this extract condition for RA treatment (Supplementary Figure S1).

**FIGURE 1:**
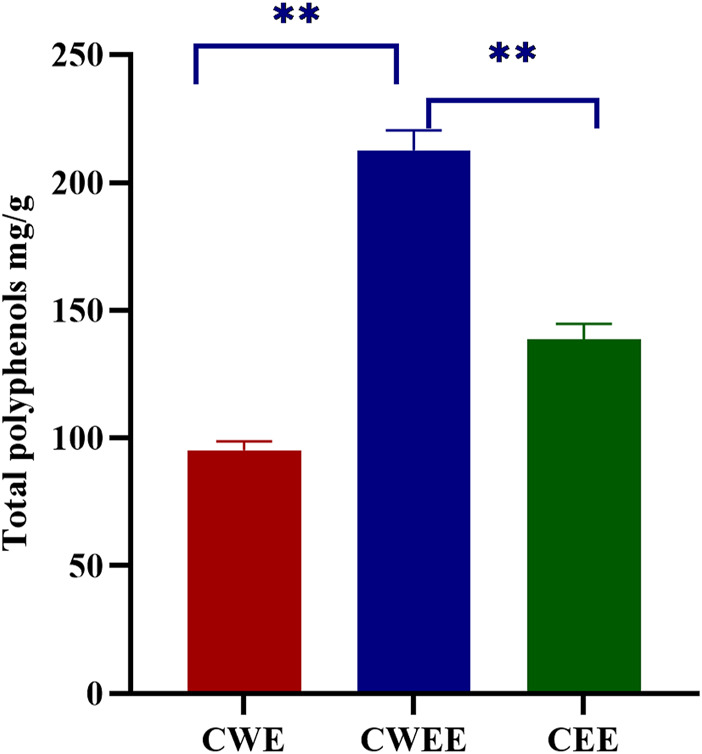
Total polyphenol content from different extracts (**p < 0.01). CWE, water extract of *Terminalia chebula*; CWEE, 50% water-ethanol extract of *T. chebula*; CEE, ethanol extract of *T. chebula*.

**FIGURE 2:**
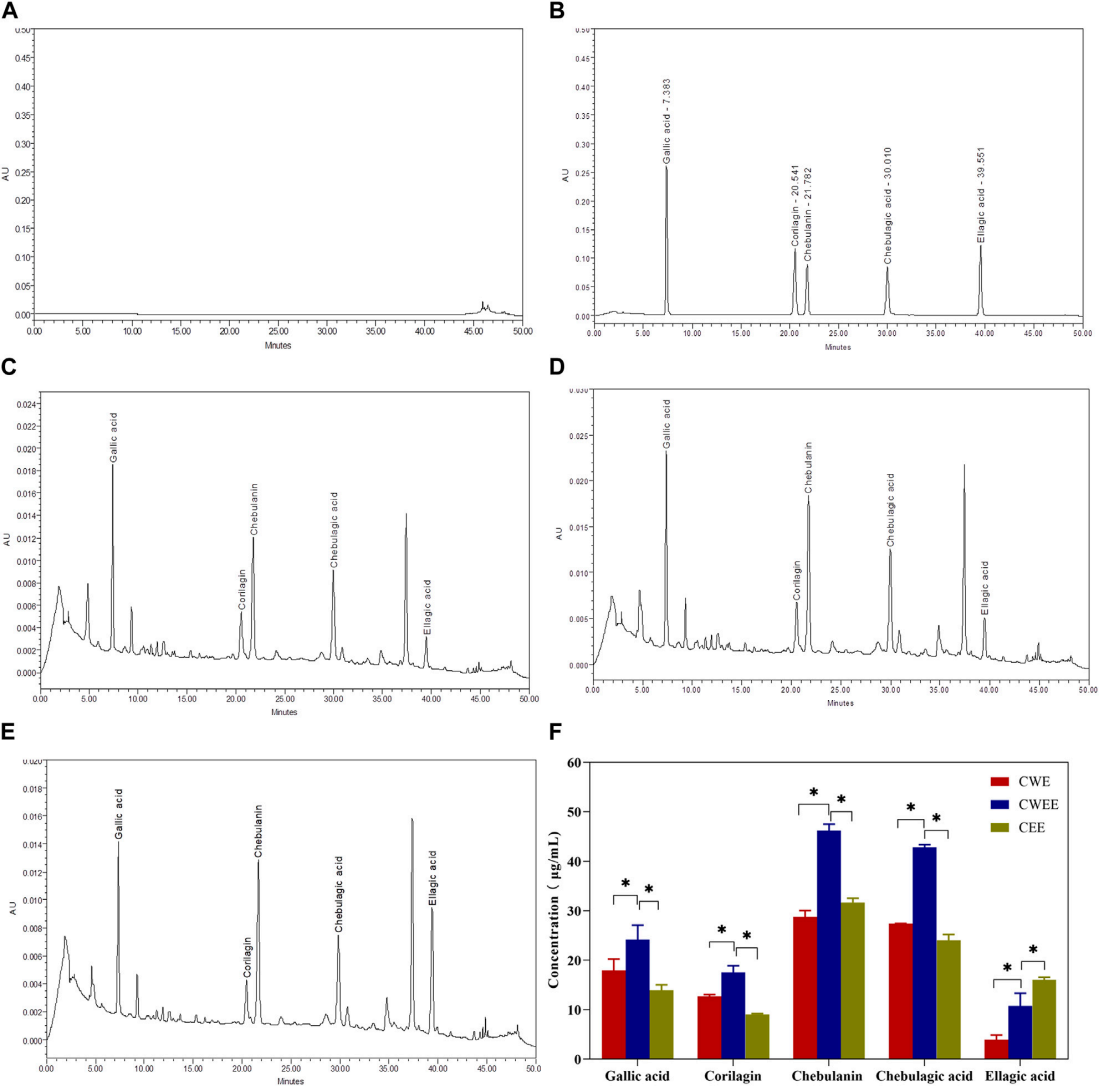
The representative chromatography of blank **(A)**, standard solution **(B)**, water extraction sample **(C)**, 50% water-ethanol extraction sample **(D)** and pure ethanol extraction sample **(E)** and quantitative analysis of the five polyphenols in different extracts **(F)**. *p < 0.05.

Next, we used the Folin–Ciocalteu reagent to identify the total polyphenol content in the fruit of *T. chebula*. The total polyphenol content in CWEE was significantly higher than that in CWE and CEE (Figure 1). Subsequently, we utilized HPLC-UV for quantitative analysis of individual polyphenols in different extracts. The representative chromatography was shown in Figure 2, We noticed that the blank sample (Figure 2A) did not interfere the determination of five polyphenols (Figure 2B). The retention time of gallic acid, corilagin, chebulanin, chebulagic acid, and ellagic acid in standard solution were 7.383, 20.541, 21.782, 30.010, and 39.551 min, respectively. The analyzed peaks in the chromatography of CWE (Figure 2C), CWEE (Figure 2D) and CEE (Figure 2E) corresponded to the respective standards in the chromatography of the standard solution.

The linearity was determined by *r* ^2^ calculated from calibration curves. The linear regression equation, concentration range, and linearity coefficients of those five polyphenols were listed in Table 1. The *r* ^2^ value of gallic acid, corilagin, chebulanin, chebulagic acid, and ellagic acid were 0.9997, 0.9996, 0.9991, 0.9991, and 0.9998, respectively. The range of gallic acid (2.23–217.56 μg/mL), corilagin (2.33–228.00 μg/mL), chebulanin (2.07–202 μg/mL), chebulagic acid (2.25–220 μg/mL), and ellagic acid (0.54–52.43 μg/mL) showed good linearity. The detailed results of accuracy, precision and stability were shown in (Supplementary Tables S1–S4). The % recovery of five polyphenols ranged from 95.2% to 103.36%, the RSD % of intra-precision ranged from 0.41% to 4.97% while the RSD % of inter-precision ranged from 1.40% to 6.16%. The stability, represented by the ratio of concentration at 12 h to concentration at 0 h of five polyphenols varied from 104.77% to 105.58%.

**TABLE 1:** Linear regression equation, linear range, and correlation coefficient of each component.

Component	Regression equation	Linear range (μg/mL)	Correlation coefficient
Gallic acid	Y = 1.29 × 10^4^ X - 4.52 × 10^3^	2.23–217.56	0.9997
Corilagin	Y = 7.24 × 10^3^ X - 3.81 × 10^3^	2.33–228.00	0.9996
Chebulanin	Y = 7.43 × 10^3^ X - 3.75 × 10^3^	2.07–202.00	0.9991
Chebulagic acid	Y = 6.63 × 10^3^ X - 4.50 × 10^3^	2.25–220.00	0.9991
Ellagic acid	Y = 8.10 × 10^3^ X - 7.51 × 10^2^	0.54–52.43	0.9998

The individual concentration of five polyphenols was exhibited in Figure 2F, it is shown that gallic acid, corilagin, chebulanin, chebulagic acid, and ellagic acid were all extracted by the three different extract solvent with varied content. Among them, CWEE showed the highest content of five polyphenols than CWE and CEE. Chebulanin was the most abundant component with concentration at 46.20 ± 1.30 μg/mL; chebulagic acid was slightly lower than chebulanin with concentration at 42.81 ± 0.56 μg/mL; subsequently followed by gallic acid with concentration at 24.18 ± 2.92 μg/mL and corilagin with concentration at 17.52 ± 1.37 μg/mL. ellagic acid showed the lowest concentration at 10.75 ± 2.60 μg/mL.

### 3.2 Antioxidant activity of different extracts and five polyphenols

**FIGURE 3:**
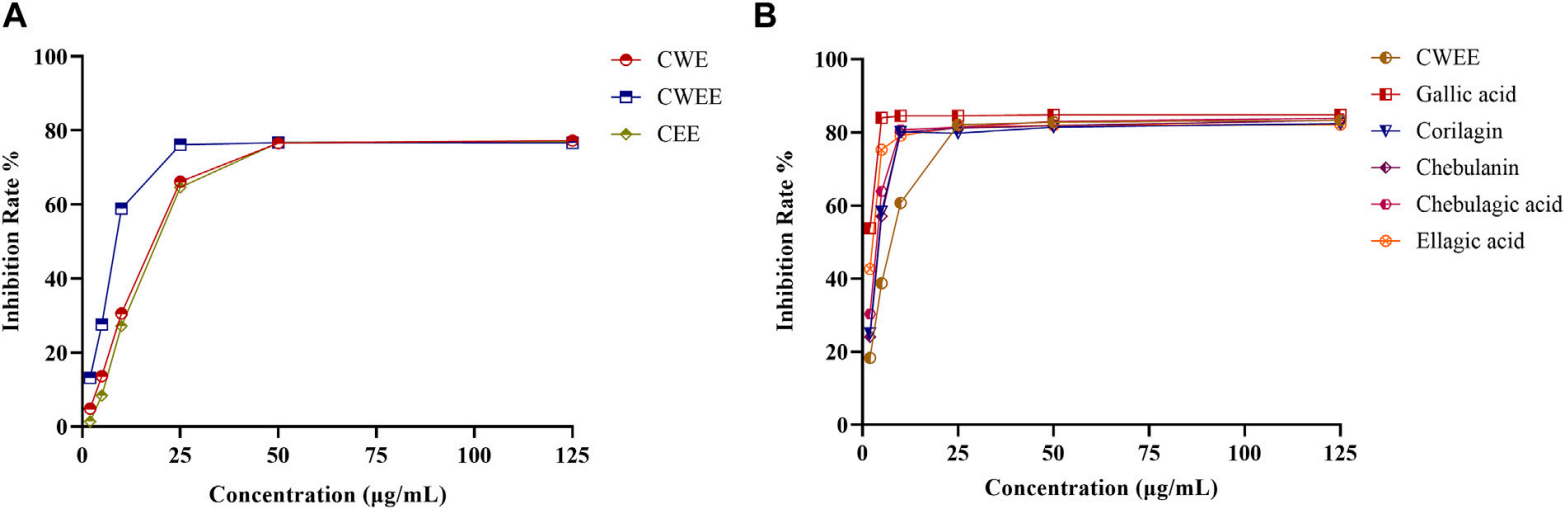
The effects of different extracts of *T. chebula* and polyphenols on DPPH radical-scavenging activity. **(A)** different extracts; **(B)** CWEE and the five polyphenol solutions.

CWEE showed the best DPPH-scavenging activity with an IC_50_ value of 7.305 μg/mL, followed by CWE with an IC_50_ value of 12.78 μg/mL and CEE with an IC_50_ value of 13.12 μg/mL (Figure 3A). The solutions of all five polyphenols showed superior antioxidant activity than CWEE. Among them, gallic acid showed the best antioxidant activity, followed by ellagic acid, and the remaining three compounds had comparable antioxidant activity (Figure 3B). All samples exhibited antioxidant activity in a concentration-dependent manner.

### 3.3 Effects of the five polyphenols from *T. chebula* on cell viability

**FIGURE 4:**
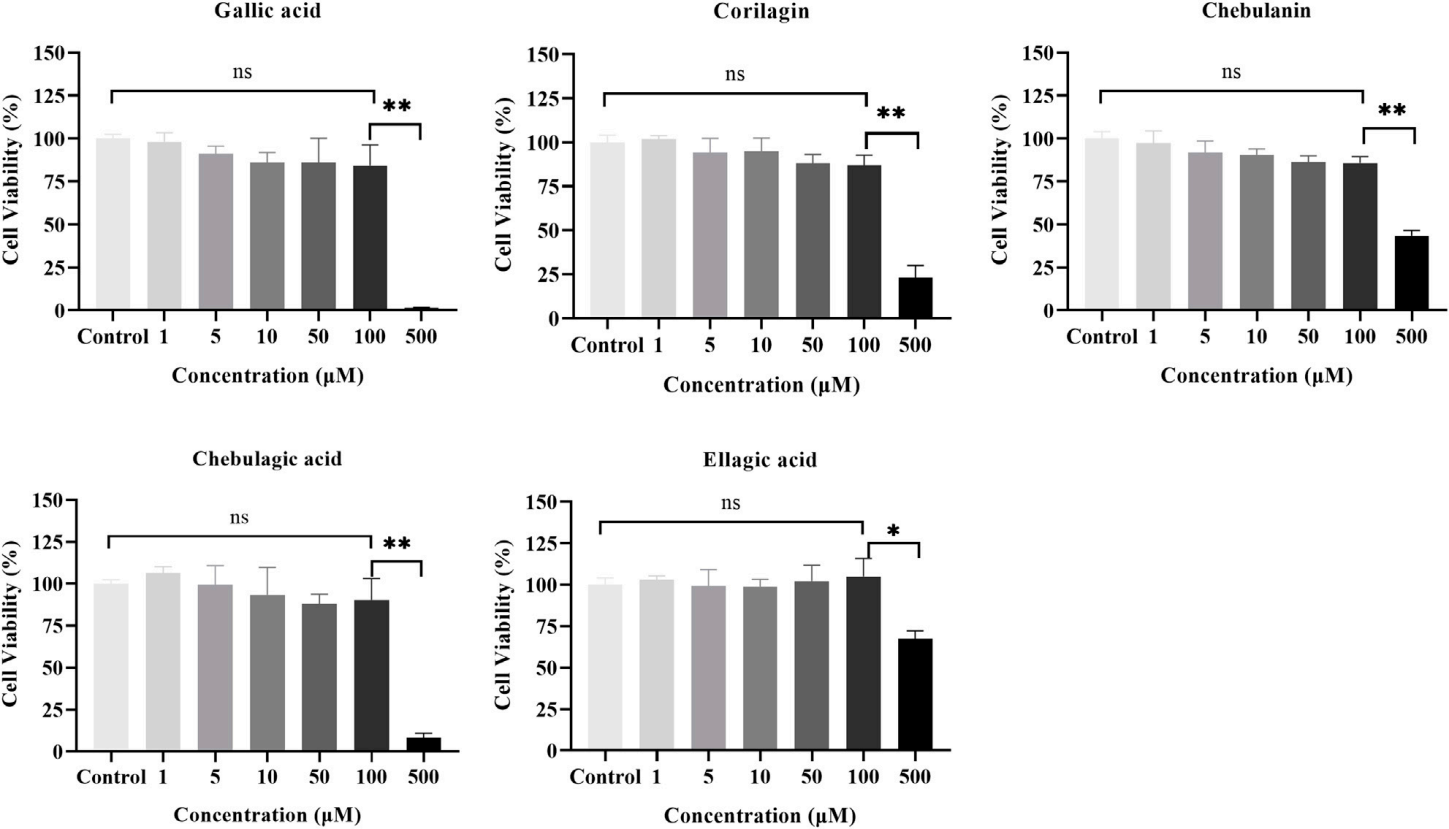
Effects of the five polyphenols on the proliferation of MH7A cells in the CCK-8 assay (*n* = 5); ns, *p* > 0.05; *p < 0.05, **p < 0.01.

We first used the CCK-8 assay to evaluate the cytotoxicity of the five polyphenols at different concentrations (0, 1, 5, 10, 50, 100, 500 μM) on MH7A cells. As shown in Figure 4, significant cytotoxic effects were observed only at 500 μM, and none of the components showed cytotoxic effects in MH7A cells at concentrations up to 100 μM; therefore, all subsequent experiments with the components were conducted with concentrations ≤100 μM.

### 3.4 Effects of the five polyphenols on pro-inflammatory cytokines in IL-1β-stimulated MH7A cells

**FIGURE 5:**
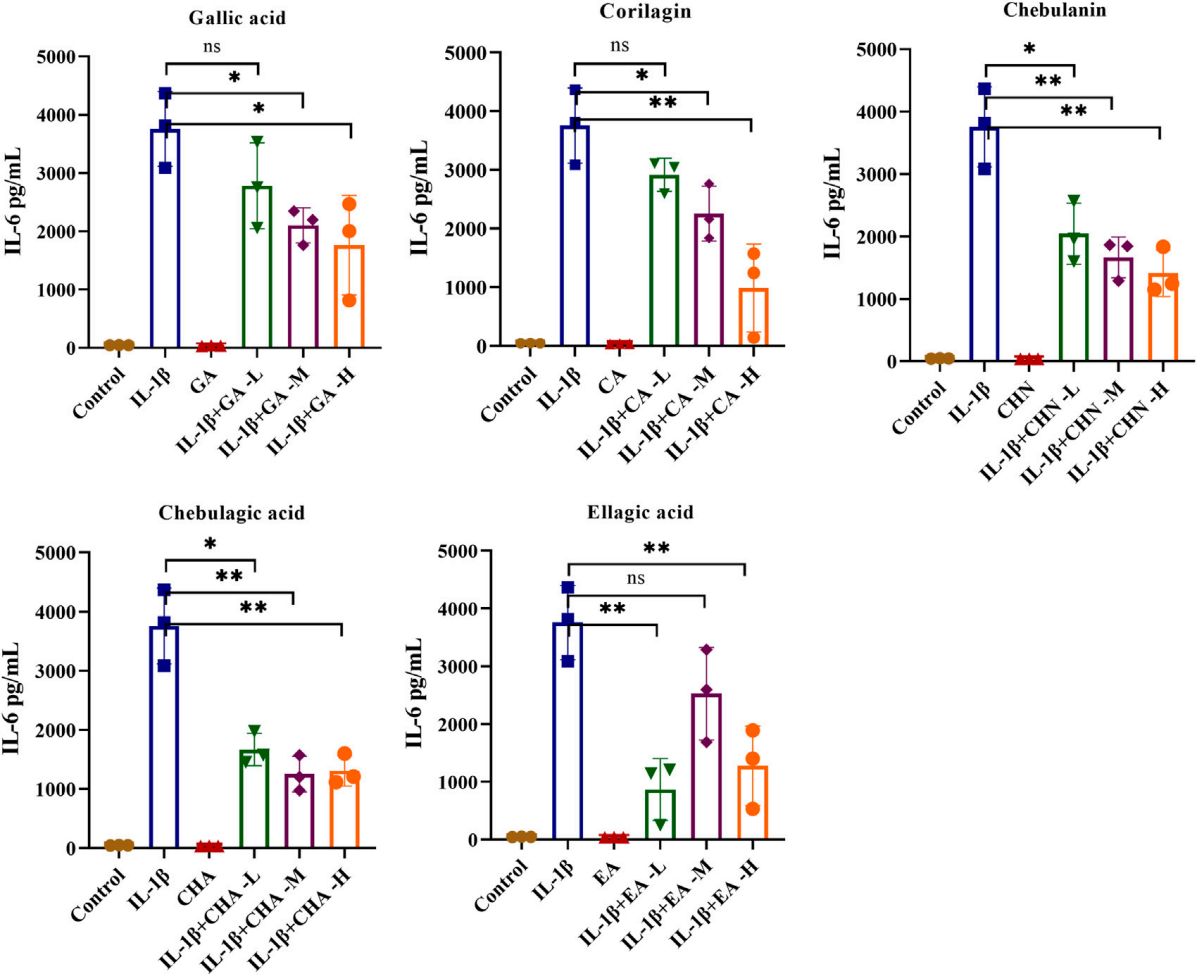
The effects of the five polyphenols on the expression of the pro-inflammatory cytokine IL-6 in IL-1β-stimulated MH7A cells (*n* = 5); ns, *p* > 0.05; *p < 0.05; **p < 0.01.

**FIGURE 6:**
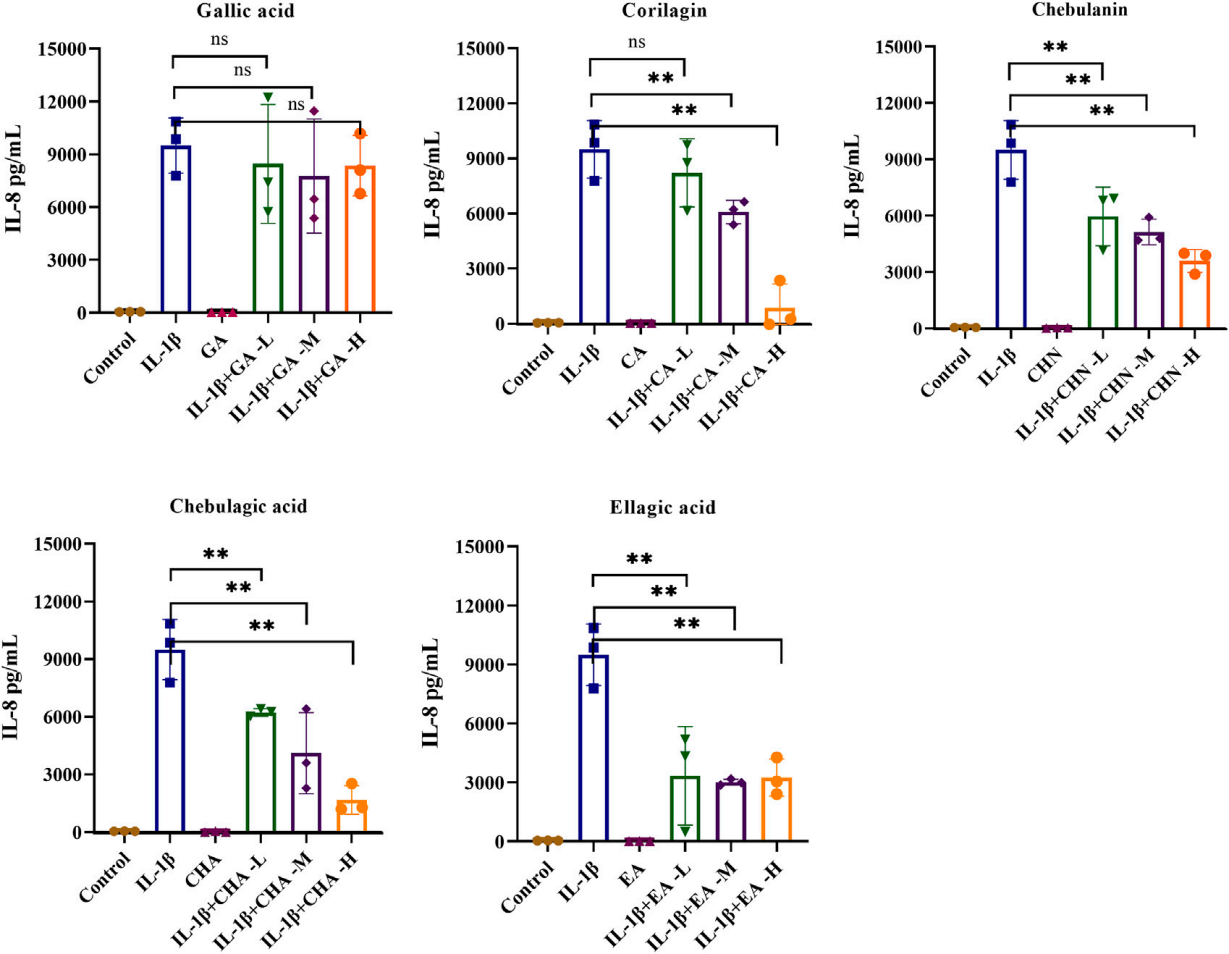
The effects of the five polyphenols on the expression of the pro-inflammatory cytokine IL-8 in IL-1β-stimulated MH7A cells (*n* = 5); ns, *p* > 0.05; *p < 0.05; **p < 0.01.

To clarify the antiarthritic activity of the five polyphenols, the levels of pro-inflammatory cytokines such as IL-6 and IL-8 in the IL-1β-stimulated MH7A cell culture supernatant were determined by ELISA. As illustrated in Figure 5, the IL-6 level was significantly increased by IL-1β stimulation, which indicated successful establishment of the immunomodulatory cell model. As expected, all five polyphenols showed a decreasing trend of IL-6 production, of which chebulagic acid showed the strongest inhibitory effect with dose-dependent inhibition rates ranging from 55.6% to 65.2%; chebulanin also showed prominent decreasing trend from 45.6% to 62.4% in a dose-dependent manner; corilagin and ellagic acid showed moderate decreasing trends, but the trends were not dose-dependent, while gallic acid showed a wild decreasing trend. The analyses of IL-8 production showed similar results for all polyphenols except gallic acid, which showed a significant reduction in IL-8 production (Figure 6), while chebulagic acid and chebulanin showed dose-dependent trends.

### 3.5 Principal component analysis clarified that chebulagic acid and chebulanin were the main active components

We used principal component analysis to calculate the scores for each polyphenol and rank them to identify the main active components. We selected the concentration of each component in the 50% ethanol extract, the IC_50_ value for DPPH-scavenging activity, and the IL-6 and IL-8 inhibitory rates as assessment indicators. After analysis, three principal components with eigenvalues greater than 1 and a cumulative variance contribution of 98.770% were extracted (Table 2).

**TABLE 2:** Explanation of the total variance.

Components	Initial eigenvalues			Extraction sums of squared loading		
	Total	% Of variance	Cumulative %	Total	% Of variance	Cumulative %
1	4.269	53.363	53.363	4.269	53.363	53.363
2	2.284	28.551	81.915	2.284	28.551	81.915
3	1.348	16.855	98.770	1.348	16.855	98.770
4	0.098	1.230	100.000			
5	5.894E^-16^	7.368E^-15^	100.000			
6	2.302E^-16^	2.878E^-15^	100.000			
7	9.315E^-16^	1.164E^-15^	100.000			
8	−4.578E^-16^	−5.722E^-15^	100.000			

Extraction method: Principal component analysis.

On the basis of the eigenvalues, the equations for calculating each principal component score (Eq. 1) and the composite score (Eq. 2) are listed below:

$$Y1=0.184X_{1}+0.319X_{2}+0.386X_{3}+0.198X_{4}+0.397X_{5}+0.429X_{6}+0.428X_{7}+0.388X_{8}$$ (1)

$$Y2=0.492X_{1}-0.382X_{2}-0.335X_{3}+0.494X_{4}-0.371X_{5}+0.180X_{6}+0.049X_{7}+0.289X_{8}$$

$$Y3=0.464X_{1}+0.409X_{2}+0.245X_{3}+0.433X_{4}+0.086X_{5}-0.320X_{6}-0.387X_{7}-0.332X_{8}$$

$$Y=0.53363\times Y_{1}+0.28551\times Y_{2}+0.16855\times Y_{3}$$ (2)

On the basis of these equations, we calculated the scores for the five components. As shown in Table 3, chebulagic acid and chebulanin showed significantly higher scores than the other three components; therefore, chebulagic acid and chebulanin were considered to be the main antiarthritic active components.

**TABLE 3:** Principal component values and comprehensive principal component values of five polyphenols.

Components	Y1	Y2	Y3	Y
Gallic acid	−3.49	0.18	0.66	−1.70
Corilagin	−0.16	0.59	−2.02	−0.26
Chebulanin	1.19	1.02	0.58	1.02
Chebulagic acid	1.71	0.86	0.73	1.28
Ellagic acid	0.75	−2.64	0.06	−0.35

## 4 Discussion

*T. chebula* has a diverse chemical composition, mainly including tannins, polyphenols, triterpenoids, flavonoids, and other components (Hassan Bulbul et al., 2022). We had previously conducted a network-based pharmacology analysis and screened 10 potential active components against RA, which mainly included polyphenols and alkaloids (Fang et al., 2019). However, since traditional Chinese medicine always shows a dose-effect relationship (Tang et al., 2012), qualitative and quantitative analysis of the chemical components are essential before *in vitro* activity investigations. In this study, we first utilized UPLC-Q-TOF high-resolution mass spectrometry for qualitative analysis of the chemical components in the extracts of *T. chebula* and identified six polyphenols, whereas four alkaloids were not detected. Therefore, the current study focused on the polyphenols found in *T. chebula*.

Water is thought to be a good solvent because of its safety and strong polarity. However, the enzyme polyphenol oxidase contained in water extracts may degrade polyphenols and reduce the biological activity in water extracts (Eid et al., 2023). Ethanol, as an organic solvent, has the potential to break down the cell wall and release more polyphenols but is less polar as compared with water (Dai and Mumper, 2010). Water is typically added to pure ethanol to increase its polarity. The use of organic solvents in conjunction with water allows for the extraction of all compounds that are soluble in both solvents, and exhibit their high extraction power (Nair et al., 2010; Bag et al., 2013). Therefore, we extracted the active components of *T. chebula* by water solvent, 50% water-ethanol solvent and pure ethanol solvent, respectively. The total polyphenol concent determined by Folin–Ciocalteu assays showed that the highest total polyphenol content was obtained with the 50% water-ethanol extracts (CWEE), followed by the water extracts (CWE), while the pure ethanol extracts (CEE) showed the lowest total polyphenol content. The results also further confirmed that the organic solvents in conjunction with water exhibit their high extraction power and is the optimal solvent for polyphenol extraction.

Subsequently, we selected HPLC to determine the levels of individual polyphenols in different extracts, and HPLC analyses again showed the highest total and individual polyphenol levels in the 50% water-ethanol extracts. Interestingly, the highest levels in our study were obtained for chebulanin, which is slightly different from the results of other studies where chebulagic acid was the predominant component (Seo et al., 2012; Manosroi et al., 2013). Previous study reported that the plant phenolic accumulation correlates with ecological growth conditions. Their portions are largely decided by abiotic conditions such as climate, meteorology (temperature, humidity), and soil composition, and also geographical factors (Mykhailenko et al., 2020). In our study, we used *T. chebula* from Guangxi Province while other studies mainly used *T. chebula* from India or Thaland. To further verify that differences in growth conditions may lead to differences in components, we purchased commercial *T. chebula* extract capsules (AyuFlex^®^; Natreon, United States) and determined the components of each polyphenol. To our surprise, gallic acid was the predominant component in this *T. chebula* extract, much higher than in the other components. We found although the active components in *T. chebula* may show significant differences in the proportions of active components but not much in terms of category when they collected from different growth condition. Based on previous reports and our current study, it is gratifying to find that extracts from *T. chebula* showed good antioxidant activity and anti-inflammatory activity with different dosage regardless of the growth condition (Seo et al., 2012; Sireeratawong et al., 2014; Yang et al., 2014; Liu et al., 2020).

Studies on polyphenols’ anti-arthritic effects have mainly focused on their influence on inflammation pathways. However, there are some studies that concentrate on the antioxidant effect (Sung et al., 2019). Furthermore, there is some evidence for a positive effect of antioxidants on clinical symptoms of RA (van Vugt et al., 2008). *T. chebula* contains large amounts of polyphenols, which show excellent antioxidant activity as reactive oxygen species (ROS) scavengers. Therefore, we first utilized the DPPH assay to evaluate the antioxidant activity of different extracts and individual polyphenols of *T. chebula*. The results showed that the water extracts, 50% water-ethanol extracts, and ethanol extracts of *T. chebula* had good antioxidant activity with IC_50_ values ranging from 7.305 to 13.12 μg/mL. The 50% water-ethanol extract showed the best scavenging activity against DPPH radicals, with an IC_50_ value of 7.305 μg/mL. The DPPH radical-scavenging activity in our study was superior to that of a previous study (Bag et al., 2013). The individual polyphenols showed better scavenging activity than the 50% water-ethanol extract. It seems likely that antioxidant activity evaluated by radical scavenging activity of polyphenols appears to depend on the number of hydroxyl groups per molecule, the degree of conjugation of galloyl units, and the number of freely rotating galloyl groups (Pfundstein et al., 2010). In this study, gallic acid and ellagic acid exhibit the highest activity on a molar basis, consistent with their high density of hydroxyls and high molecular mobility. The corilagin, chebulanin and chebulagic acid exhibit a little weak antioxidant activity may due to the less freely rotating groups. It has been reported PI3K/Akt pathway which produces heme oxygenase-1 through transcription of the Nrf2 gene is thought to be the key element in the oxidative pathway that attributed a role in the reduction of RA symptoms by polyphenols (Sung et al., 2019). Gallic acid (Lin et al., 2020) and ellagic acid (Zhu et al., 2022) may reduce oxidative stress by enhancing Nrf2 signaling pathway and alleviate arthritis.

RA is characterized by inflammation of the synovial membrane and proliferation of the synovial lining. Fibroblast-like synoviocytes and macrophage-like synoviocytes play critical roles in the destructive process of RA (Tu et al., 2018). In the early stage of RA development, activated macrophages secrete pro-inflammatory cytokines such as TNF-α, IL-1β and IL-6. Such pro-inflammatory cytokines subsequently activate fibroblast-like synoviocytes. Fibroblast-like synoviocytes synthesizes and secretes mediators of inflammation including IL-6 and IL-8 (Georganas et al., 2000). Furthermore, a large body of literature has proposed that IL-6 and IL-8, two pro-inflammatory factors were generally chosen as pro-inflammatory cytokines (Li et al., 2014; Mijiti et al., 2021). Therefore, in this study, IL-1β-induced MH7A cells were selected to construct an *in vitro* inflammation model to examine the effects of the polyphenols on the pro-inflammatory factors IL-6 and IL-8. The results showed some variability in the antioxidant results. Gallic acid had the strongest antioxidant activity but showed no significant inhibitory effect on IL-8 and a weak inhibitory effect on IL-6. The antioxidant activity is mainly related to the phenolic hydroxyl groups present in the compounds, with more phenolic hydroxyl groups corresponding to stronger antioxidant activity. The anti-inflammatory activity was not significantly correlated with the phenolic hydroxyl groups.

Polyphenols primarily regulate the inflammatory pathway *via* the MAPK and NF-κB signaling pathways (Sung et al., 2019). In RA synovial tissue, the transcription factor NF-κB is activated. Binding to this transcription factor’s site has been identified in the promoter regions of the IL-6 and IL-8 genes, activating both pro-inflammatory genes (Georganas et al., 2000). Previous research found that chebulanin and chebulagic acid, which reduced IL-6 and IL-8 expression, also inhibited the NF-B signaling pathway and the MAPK signaling pathway (Reddy and Reddanna, 2009; Liu et al., 2020). Our previous network pharmacology analysis revealed that the NF-κB signaling pathway and MAPK signaling pathway play dominate role in the mechanism of *T. chebula* for RA treatment (Fang et al., 2019). Other studies are consistent with our findings (Ekambaram et al., 2022). The structural analysis revealed that both chebulanin and chebulagic acid contain 2,4-O-chebuloyl-β-d-glucose gallic acyl derivative structural domains, which are presumed to be key domains for anti-inflammatory activity. However, further studies are needed to validate the importance of this domain.

Our study, however, has some limitations. Six polyphenols were identified through network pharmacology analysis: gallic acid, chebulic acid, corilagin, chebulanin, chebulagic acid, and ellagic acid. We only quantified five polyphenols except for chebulic acid. It’s because the standard chebulic acid product was not available on the market when we conducted this study. When the standard product became available, we purchased it and evaluated its anti-arthritic effect first. However, chebulic acid was found to have the least anti-arthritic activity of the six polyphenols studied. As a result, we did not include it in our study.

## 5 Conclusion

Taken together, we successfully established a rapid, accurate, and convenient HPLC method for the quantitative analysis of the polyphenols gallic acid, corilagin, chebulanin, chebulagic acid, and ellagic acid in *T. chebula*. Among these components, chebulanin and chebulagic acid were the most abundant. Furthermore, our findings revealed the potential role of chebulanin and chebulagic acid from *T. chebula* as anti-arthritic drugs.

## Data Availability

The original contributions presented in the study are included in the article/Supplementary Material, further inquiries can be directed to the corresponding authors.
