# Supplementary material for: Simultaneous quantitative analysis and in vitro anti-arthritic effects of five polyphenols from Terminalia chebula
Source: Front Physiol. 2023 Mar 8;14:1138947. doi: 10.3389/fphys.2023.1138947 (PMC10030958; doi:10.3389/fphys.2023.1138947)
Supplement: Supplementary file 1 [file DataSheet1.pdf]

Supplementary figures and tables

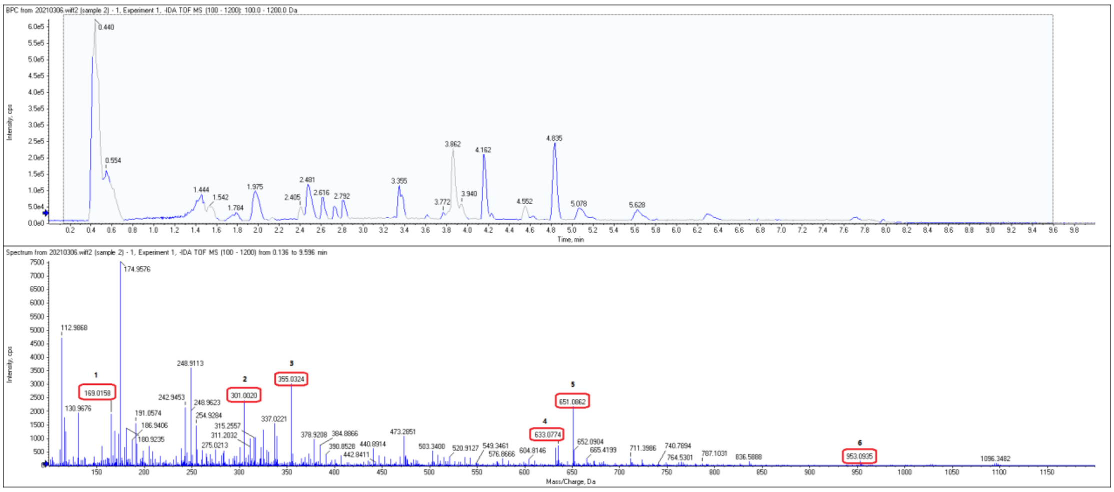

Supplementary Figure 1 The TIC chromatography of *Terminalia chebula* extraction in negative model with ESI in UPLC-Q-TOF（1-Gallic acid；2-ellagic acid；3-chebulic acid；4-corilagin；5-chebulanin；6-chebulagic acid）

Supplementary Table 1 the recovery results of five polyphenols in low, middle and high concentrations (n=5)

	Low concentration（μg/mL）			Middle concentration（μg/mL）			High concentration（μg/mL）		
	estimate	calculate	recovery %	estimate	calculate	recovery %	estimate	calculate	recovery %
gallic acid	5.57	5.57±0.10	99.98±1.76	34.81	33.87±0.14	97.31±0.40	217.56	219.20±1.67	100.76±0.77
corilagin	5.84	5.86±0.15	100.38±2.63	36.48	35.55±0.24	97.44±0.67	228.00	230.63±2.27	101.15±1.00
chebulanin	5.17	5.10±0.14	98.56±2.75	32.32	31.01±0.96	95.94±2.97	202.00	202.54±4.43	100.27±2.19
chebulagic acid	5.63	5.51±0.27	97.78±4.86	32.20	33.51±1.20	95.20±3.41	220.00	219.70±6.60	99.86±3.00
ellagic acid	1.34	1.35±0.05	100.69±3.40	8.39	8.29±0.06	98.85±0.73	52.43	54.19±1.07	103.36±2.03

Supplementary Table 2 The intra-day precision results of five polyphenols in low, middle and high concentrations (n=5)

	Low concentration（μg/mL）			Middle concentration（μg/mL）			High concentration（μg/mL）		
	estimate	calculate	RSD%	estimate	calculate	RSD%	estimate	calculate	RSD%
gallic acid	5.57	5.57	1.76	34.81	33.87	0.41	217.56	219.20	0.76
corilagin	5.84	5.86	2.62	36.48	35.55	0.68	228.00	230.63	0.98
chebulanin	5.17	5.10	2.79	32.32	31.01	3.10	202.00	202.54	2.19
chebulagic acid	5.63	5.51	4.97	32.20	33.51	3.58	220.00	219.70	3.00
ellagic acid	1.34	1.35	3.38	8.39	8.29	0.74	52.43	54.19	1.97

Supplementary Table 3 The inter-day precision results of five polyphenols in low, middle and high concentrations (n=15)

	Low concentration（μg/mL）			Middle concentration（μg/mL）			High concentration（μg/mL）		
	estimate	calculate	RSD%	estimate	calculate	RSD%	estimate	calculate	RSD%
gallic acid	5.57	5.59	2.88	34.81	34.48	1.50	217.56	220.30	1.40
corilagin	5.84	5.84	2.93	36.48	36.16	1.48	228.00	231.52	1.48
chebulanin	5.17	5.04	2.61	32.32	31.41	2.02	202.00	203.53	1.96
chebulagic acid	5.63	5.46	4.11	32.20	34.12	2.40	220.00	221.23	2.41
ellagic acid	1.34	1.35	4.45	8.39	8.63	4.99	52.43	56.39	6.16

Supplementary Table 4 The stability of five polyphenols in sample as placed in the sample tray for 12h (n=3)

	0h	12h	Concentration ratio
gallic acid	4.09±0.13	4.31±0.01	105.55
corilagin	4.98±0.15	5.26±0.04	105.58
chebulanin	18.75±0.58	19.69±0.09	104.99
chebulagic acid	12.82±0.37	13.43±0.08	104.74
ellagic acid	10.84±0.34	11.41±0.04	105.34
